# Influence of Nanoencapsulation Using High-Pressure Homogenization on the Volatile Constituents and Anticancer and Antioxidant Activities of Algerian *Saccocalyx satureioides* Coss. et Durieu

**DOI:** 10.3390/molecules25204756

**Published:** 2020-10-16

**Authors:** Abdelhakim Aouf, Hatem Ali, Abdel Rahman Al-Khalifa, Khaled Fahmy Mahmoud, Amr Farouk

**Affiliations:** 1 Laboratory of Applied Microbiology, Faculty of Life Sciences and Nature, University of Ferhat Abbas, Sétif-1, Sétif 19000, Algeria; a.aouf@univ-setif.dz; 2 Food Science and Nutrition Department College of Food Science and Agriculture, King Saud University, Riyadh 12372, Saudi Arabia; haali@ksu.edu.sa (H.A.); akhalifa@ksu.edu.sa (A.R.A.-K.); 3 Food Technology Department, National Research Centre, Cairo 12622, Egypt; khaledfm69@yahoo.com; 4 Flavour and Aroma Chemistry Department, National Research Centre, Cairo 12622, Egypt

**Keywords:** *Saccocalyx satureioides*, essential oil, high-pressure homogenization, nanoemulsion, MTT, antioxidant

## Abstract

The nanoencapsulation of essential oils enhances their applicability in several areas, such as pharmaceuticals and food biopreservation. This study focuses on the encapsulation of *Saccocalyx satureioides* Coss. et Durieu essential oil into nanoemulsions by high-pressure homogenization (HPH) and its effect on the volatile constituents and the antioxidant and anticancer activities of the essential oil. The analysis of hydrodistilled (HD) *S. satureioides* essential oil using gas chromatography–mass spectrometry revealed a total of 28 constituents, representing 99.80%, while only 13 constituents were identified in nanoemulsions, representing 98.65% of the total volatile material. The use of HPH led to qualitative and quantitative differences between the volatile profiles of the HD and the nanoemulsion of *S. satureioides* essential oil. Whereas borneol, α-terpineol, and thymol were the predominant constituents in the HD oil, carvacrol, thymol, and γ-terpinene were the major constituents in the nanoemulsion. The antioxidant activity of the *S. satureioides* essential oil nanoemulsion displayed was lower as compared to that of HD oil using DPPH free radical–scavenging, CUPRAC, and ABTS assays. This is consistent with the differences in total flavonoid, total phenolic, and volatiles detected in both HD oil and its nanoemulsion. Meanwhile, the cytotoxicity on liver cancer cells (Hep-G2) was stronger using nanoemulsions (106 μg/mL) than using HD oil (274.8 μg/mL).

## 1. Introduction

Knowledge in medicinal plants is growing exponentially through the determination of the chemical compositions and biological activities of thousands of species. This knowledge is now exploited in thousands of applications ranging from human and veterinary medicines, agriculture, food biopreservation, ingredients of fragrances, flavoring, and decorative cosmetics [1,2]. The Lamiaceae family, with 236 genera and more than 7000 species, is known for its aromatic members. This family has a cosmopolitan distribution, while the genus *Saccocalyx satureioides* Coss. et Durieu does not belong to the largest genera of this family. It was reported only in northeastern Algeria, and it is endemic, particularly in a pre-desert area [3]. It was first described in 1835 [4]. The impressive results obtained from studies on the chemical composition of its essential oils and their biological activities revealed that this plant has earned an important place among medicinal plants [3,5]. A review of the literature showed that the complex mixture of the bioactive compounds of its essential oil includes the following components: borneol, thymol, α-terpineol, and p-cymene [5]. In addition to its uses in folk medicine to treat spasms and gastric disorders, this wealth of active compounds encourages researchers to apply it in various fields as antimicrobial, antifungal, and an antioxidant for the oil extracted from the plant cultivated in Djelfa, Algeria [3].

Applications of essential oils including *Saccocalyx satureioides* Coss. et Durieu oil is currently limited in the pharmaceutical and food industries owing to their sensitivity to and instability under environmental stress, as well as their volatility, aroma, and flavor. The former drawbacks make encapsulation into nanoemulsion an attractive technique that may facilitate the use of different essential oils in more fields [2,6]. The essential oil is more stable when stored as nanoemulsion vesicles with a droplet size range of 20 to 200 nm. The reduction of a droplet size is a comminuting process based on energy-intensive techniques, e.g., high-pressure homogenization (HPH), which enables the breakup of the bulk oil phase with the formation of nano-oil droplets dispersed in the water phase [7].

Therefore, this study evaluated the effect of nanoemulsions using HPH techniques on the volatile constituents, antioxidant activities, and cytotoxic effects on liver cancer cell line Hep-G2 of the *S. satureioides* Coss. et Durieu essential oil obtained by hydrodistillation. Healthy human hepatic cells (THLE2) were used as a control to examine the assumed selectivity of the examined oil and its nanoemulsion. The investigation of safe materials of botanical origin in this study represented a flourishing opportunity for applying nanotechnology and essential oils in various food and pharmaceutical industries.

## 2. Results and Discussion

### 2.1. Effect of Encapsulation Using HPH on the Volatile Oil of S. satureioides Coss. et Durieu

**Figure 1:**
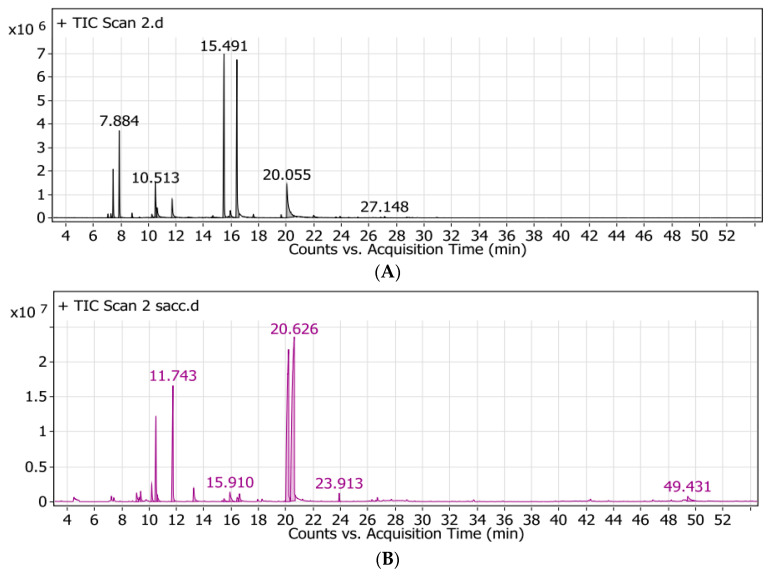
Volatile chromatograms for (**A**) *S. satureioides* HD oil and (**B**) nanoemulsions of *S. satureioides* oil.

The dried aerial parts submitted to hydrodistillation produced a yellow oil with a yield of 2.1 ± 0.05%. Bendimerad et al. also reported similar results [8]. The identification of chemical composition using gas chromatography–mass spectrometry (GC–MS) revealed the presence of 28 constituents, representing 99.80% of the total hydrodistilledessential oil (HD) content (Table 1, Figure 1A). Borneol and α-terpineol were the predominant compounds with 25.71% and 25.61%, respectively, followed by thymol (12.8%), camphene (10.26%), α-pinene (5.68%), p-cymene (5.61%), and γ-terpinene (3.89%). From the qualitative and quantitative points of view, these results are in line with the findings of Laouer et al. [9] but they differ quantitatively when compared to those of Biondi et al. [4], Bendahou et al. [5], and Achraf et al. [10]. Conversely, thymol was detected with a large predominance in comparison with borneol and α-terpineol. This could be due to endogenous and exogenous factors such as individual genetic variability, differences in growing locations, agronomical practices, and environmental conditions that were reported as the main reasons affecting the chemical composition of essential oils [11,12].

The findings of chemical analysis of the oil nanoemulsions by GC–MS showed a great difference from that of the HD oil. Only 13 constituents were identified in nanoemulsions, representing 98.65% of the total nanoemulsion oil. Like HD oil, oxygenated monoterpenes were predominant but with quantitative distinctions, carvacrol (41.27%) and thymol (29.99%) were predominant, while borneol and α-terpineol were detected in minor amounts in comparison with their concentrations in HD oil (0.41% and 1.60%, respectively) (Table 1, Figure 1B). Some of the non-oxygenated terpenes, e.g., α-terpenine and γ-terpinene, were increased in the oil nanoemulsion, whereas other predominates, such as α-pinene and camphene, were not detected in comparison with the HD oil. Most of the studies dealt with the encapsulation of oils or flavors and focused on the physical stability and biological activity of the microparticles or nanoparticles but not on the changes in the volatile constituents of the encapsulated oils. Few studies have reported that the formulation based on energy-intensive techniques may lead to Ostwald ripening, flocculation, or coalescence of the emulsion with changes in its physical stability and biological activity [13]. HPH and high shear homogenization represent examples of energy-intensive techniques that result in the decomposition of the active constituents of essential oils and the accumulations of others [14,15]. To the best of our knowledge, nothing has been reported about the effect of encapsulation techniques on chemical changes among the divergent terpenes of essential oils or flavors. Interestingly, the large predominance of thymol and carvacrol in the oil nanoemulsion was at the expense of borneol and α-terpineol concentrations detected in the HD oil (Table 1). Meanwhile, an inverse relationship was reported by Ali et al. [15], while borneol and α-terpineol were reported as predominates in the nanoemulsion during the encapsulation of the *Origanum glandulosum* Desf. oil. Therefore, further studies can be conducted to examine the stability of different aroma and volatile compounds during microencapsulation techniques, especially under severe conditions, and discover the mechanisms of the transfer of such volatile compounds to others.

### 2.2. Nanoemulsion Particle Morphology

**Figure 2:**
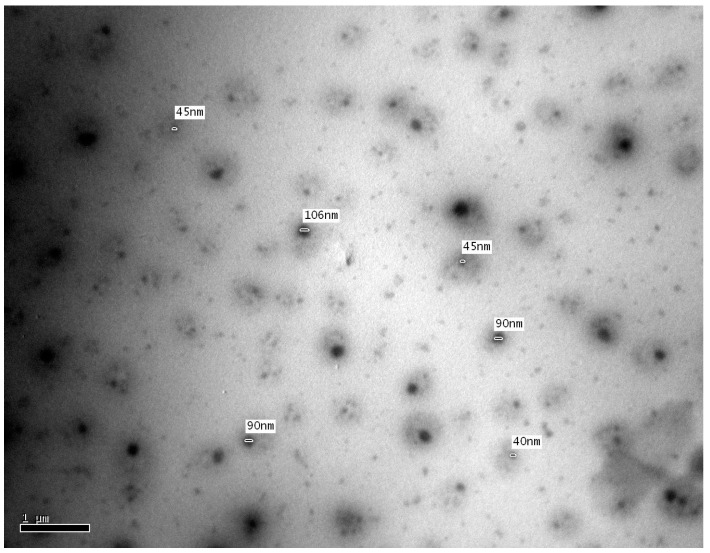
Transmission electron microscope (TEM) image of *S. satureioides* oil nanoemulsions.

The transmission electron microscope (TEM) characterization of oil nanoemulsions gives the actual size and shape, and droplets in nanoemulsions appear dark. The TEM micrograph showed that the nanoparticles were spherical in shape and had an average diameter of 68.4 ± 0.13 nm based on ImageJ software that adjusted the contrast between background and particles and calculated the diameter of rounded nanoparticles automatically (Figure 2). Evidently, the average diameter of the nanoparticles detected in this study was higher than that reported for comparable oils under the same conditions [15]. This could be related to the Ostwald ripening phenomenon where the oil phase exhibited a mild solubility in the surrounding aqueous phase of the emulsion system with transfer from small to large droplets [17].

### 2.3. Effect of Encapsulation Using HPH on the Antioxidant Activity of S. satureioides Coss. et Durieu HD Oil

Many diseases, such as cancer, liver disease, and Alzheimer’s disease, result from oxidative damage. The ability of antioxidants to prevent oxidative damage made from antioxidant activity test is one of the most intensively studied topics [18,19]. The DPPH (2,2′-diphenyl-1-picrylhydrazyl) free radical–scavenging, CUPRAC (Cupric reducing antioxidant capacity), and ABTS (2,2′-azino-bis(3-ethylbenzothiazoline-6-sulfonic acid) cation radical decolorizationassays are widely used for the evaluation of plant antioxidant activity and are sensitive in detecting active compounds, even at very low concentrations [20]. These techniques were implemented to evaluate the antioxidant activity of the *S. satureioides* Coss. et Durieu HD oil and its nanoemulsions. The findings revealed that the antioxidant activity of the HD oil of *S. satureioides* was high with the lowest IC_50_ and A_0.5_ values (22.14, 37.72, and 11.39 µg/mL) and that the antioxidant activity of the nanoemulsions was relatively low with higher IC_50_ (47.87, 754.67, and 257.86 µg/mL) towards DPPH free radical–scavenging, CUPRAC, and ABTS assays, respectively, compared with TROLOX and ascorbic acid which were used as positive control (Table 2). This finding is consistent with the only study found recently in this field examining the antioxidant activity of the *S. satureioides* oil at different harvest periods and extracted from plants cultivated in Djelfa, Algeria [3]. Due to the important role of phenolic and flavonoid compounds in exhibiting antioxidant effects, the total contents of both classes were analyzed. The HD oil had the highest phenolic and flavonoid contents and therefore exhibited more effective radical scavengers than nanoemulsions. The findings of this study showed that the major constituents of HD oils and their nanoemulsions are oxygenated monoterpenes, and they are especially known for their antioxidant activity with respect to the synergistic interaction between the phenolic content and other components in the essential oil, which was possibly responsible for the difference in the activity observed [21,22]. The antioxidant activity of oxygenated monoterpenes, including thymol, carvacrol, α—terpineol, borneol, terpene-4-ol, and linalool detected in the present study is due to the presence of phenolic nuclei (Table 1). Mohamadi et al. [23] identified 17 phenolic compounds and flavonoids during the investigation of chloroform, butanol, and ethyl acetate extracts of *S. satureioides* Coss. et Durieu using liquid chromatography, whereas caffeic acid and quercetin were the most active components of different antioxidant assays applied.

### 2.4. Effect of Encapsulation Using HPH on the Anticancer Activity of S. satureioides Coss. et Durieu HD Oil

**Figure 3:**
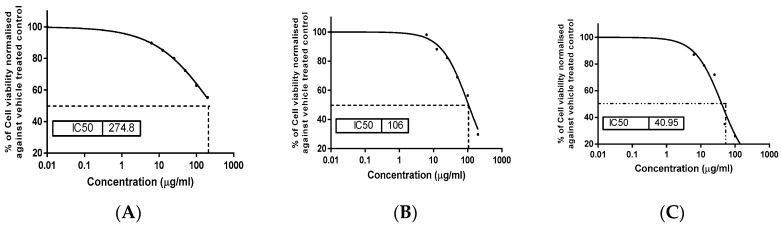
Evaluation of cell viability percentage of liver cancer cell line (Hep G2) posttreatment: (**A**) *S. satureioides* HD oil and (**B**) nanoemulsions of *S. satureioides* oil compared with reference drug (**C**) 5-flurouracil using MTT(tetrazolium bromide solution) assay.

**Figure 4:**
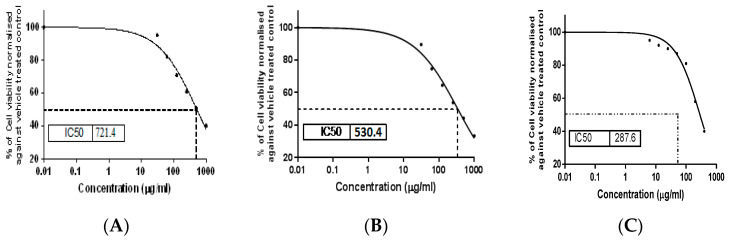
Evaluation of cell viability percentage of Healthy human hepatic cells(THLE2) posttreatment: (**A**) *S. satureioides* HD oil and (**B**) nanoemulsions of *S. satureioides* oil compared with reference drug (**C**) 5-flurouracil using MTT assay.

The MTT (tetrazolium bromide solution) viability assay was used to investigate the in vitro cytotoxic activity of the *S. satureioides* HD oil and its nanoemulsions on Hep G-2 and THLE2 cell lines in comparison with 5-fluorouracil (5-FU) as a reference drug. Figure 3 and Figure 4 summarize the results. Generally, HD oils and their nanoemulsions showed some degree of cytotoxicity against the studied cells. This is revealed by a reduction in the cell viability percentage of the liver cancer cell line (Hep G-2) after treatment in comparison with THLE2 cells, which indicated a selectivity of the studied HD oil and its nanoemulsions. Oil nanoemulsions exhibited the highest growth inhibitory activity against the Hep G-2 cell line with the lowest IC_50_ (106 μg/mL) compared with the HD oil (274.8 μg/mL). However, both were lower than the reference drug, 5-Fu (IC_50_ 40.95 μg/mL).

Table 1 shows that the antiproliferative effect is mainly due to bioactive mono- and sesquiterpenes, especially the oxygenated ones that represent the major components identified in the *S. satureioides* HD oil and its nanoemulsions, such as p-cymene, γ-terpinene, borneol, α-terpineol, thymol, and carvacrol [24,25]. The potential efficacy of the examined *S. satureioides* HD oil and its nanoemulsions is believed to be based on the cytotoxicity of their main active components and/or their synergic effect. However, the quantitative differences recorded among these components could be responsible for the variation in the cytotoxic activity between the HD oil and its nanoemulsions. According to Elshafie et al. [26], the cytotoxic activity of the predominate components in *Origanum Vulgare* L. (carvacrol, citral, and thymol) was higher than that of the crude oil itself, which was also reported by Ozkan and Erdogan [21]. Moreover, p-cymene and its metal complexes were reported as antitumor agents with inhibiting tumor proliferation through an antiangiogenic mechanism, cancer cell cytotoxicity, and anti-adhesion [27], while γ-terpinene recorded a mild cytotoxic activity in divergent tested cell lines [28]. Hence, the higher cytotoxicity of the *S. satureioides* oil nanoemulsions could be due to the higher content of carvacrol, thymol, and p-cymene in comparison with the HD oil regardless of the total phenolic and flavonoid contents (Table 1). Several mechanisms were reported to reveal the cytotoxicity effect of active constituents, such as thymol and carvacrol [26]. The induction of glutathione S-transferase activity in various tissues to eliminate chemical carcinogens is one of those mechanisms [29]. Another possible mechanism is based on the induction of cell death by apoptosis and/or necrosis, which leads to increasing the cell permeability and hence losing several cytoplasmic organelles [30,31]. The findings of the study revealed that the synergetic and/or antagonistic effect of the main constituents of the HD oil and its nanoemulsions constitute the main factor in assessing their cytotoxic activity and that the engagement between anticancer and antioxidant activities needs further investigation.

## 3. Materials and Methods

### 3.1. Plant Material and Chemicals

The aerial parts of *Saccocalyx satureioides* Coss. et Durieu were collected in March 2018 in Bousaada, M’Sila Province located between el-Hodna Salt Lake and Saharan Atlas Mountains (north-central of Algeria). Thereafter, they were dried in an obscure and dry place at ambient temperature. The genus was authenticated by a taxonomist at the laboratory of Botany, Faculty of Sciences, Ferhat Abbas University, Sétif-1. Diethyl ether and methanol were obtained from Fisher Chemicals (Pittsburgh, PA, USA). The mixture of n-alkanes (C_6_–C_26_), authentic compounds, sodium bicarbonates, linoleic acid (≥99%), Tween 20, Folin-Ciocalteu reagent, 2,2′-diphenyl-1-picrylhydrazyl (DPPH), 2,22—azino-bis-3-ethylbenzthiazoline-6-sulphonic acid (ABTS, purity >99%), methanol, potassium persulfate, neocuproine, copper(II) chloride, ammonium acetate, rutin, aluminum chloride, (+)-catechin, TROLOX ((±)-6-Hydroxy-2,5,7,8-tetramethylchromane-2-carboxylic acid), ascorbic acid, 5-fluorouracil (5-FU), and tetrazolium bromide solution(MTT) were purchased from Sigma Aldrich Chemical Co. (St. Louis, MO, USA). The human hepatocellular carcinoma (HepG2) and normal liver (THLE2) cells were purchased from the VACSERA (Cairo, Egypt), and the DMSO was provided by Merck, Darmstadt, Germany. The fetal calf serum (FCS) and penicillin/streptomycin were obtained from Hyclone, Logan, UT, USA, and Dulbecco’s, Modified Eagle Medium (DMEM) was purchased from Gibco; Thermo Fisher Scientific, Inc., Waltham, MA, USA.

### 3.2. Extraction of Essential Oil

The extraction of essential oils from the dried aerial parts of *S. satureioides* Coss. et Durieu was conducted by hydrodistillation for 3 h using a Clevenger-type apparatus. The extracted essential oils were dried using anhydrous sodium sulfate and stored in airtight glass vials covered with aluminum foil at −20 °C until analysis [12]. The experiment was performed in triplicate.

### 3.3. Preparation of S. satureioides Coss. et DurieuEssential Oil Nanoemulsions

The primary emulsions were obtained by mixing 1 g of essential oil and 1% of Tween 20 in 100 mL of deionized water using an Ultra Turrax T25 (IKA Labortechnik, Germany) at 18,000 rpm for 10 min. The obtained emulsions were subjected to high pressure homogenization (HPH) in a nano DeBEE electric bench-top laboratory homogenizer (BEE International, South Easton, MA 02375, USA) ten times at 350 MPa. At the end of HPH, hot nanoemulsions were rapidly cooled in an ice bath to crystallize oil droplets [15].

### 3.4. Gas Chromatography–Mass Spectrometry (GC–MS)

The analysis of volatile constituents from the HD and nanoemulsions of *S. satureioides* essential oil was performed using a GC–MS apparatus. A trace GC ultra-chromatography system equipped with an ISQ-mass spectrometer and a 60 m × 0.25 mm × 0.25 μm-thick TG-5MS capillary column (Thermo Scientific, Waltham, MA, USA) was programmed from 50 °C with a holding time of 3 min, and then the temperature was increased at a rate of 4 °C per min to 140 °C with a holding time of 5 min. Consequently, the temperature was increased at a rate of 6 °C per minute to 260 °C for a 5-min isothermal holding time. The injector temperature was 180 °C, the ion source temperature was 200 °C, and the transition line temperature was 250 °C. The carrier gas was helium with a constant flow rate of 1.0 mL min^−1^. The mass spectrometer had a scan range from m/z 40–450, and the ionization energy was set at 70 Ev. The identification of compounds was based on matching with the MS computer library (NIST library, 2005 version) and comparison with those of authentic compounds and published data [3,4,5,10,16]. The relative percentage of the identified constituents was calculated from the GC peak areas, and Kovats index was calculated for each compound using the retention times of a homologous series of C_6_–C_26_ n-alkanes and by matching with the literature [16].

### 3.5. Transmission Electron Microscopy (TEM)

Transmission electron microscopy (JED 1230, JEOL Ltd., and Tokyo, Japan) was employed to characterize nanoemulsions. Twenty microliters of diluted samples were placed on a film-coated 200-mesh copper specimen grid for 10 min, and the excess fluid was eliminated using a filter paper. The grids were then stained with one drop of 3% phosphotungstic acid and allowed to dry for 3 min. The coated grids were dried and examined under the TEM microscope. The samples were also observed by operating at 160 kV, while the average particle size was calculated from TEM images using the ImageJ program [32].

### 3.6. Antioxidant Activity Measurements

DPPH radical–scavenging assay:In this spectrophotometric method, DPPH was used as a reagent to measure the DPPH free radical–scavenging activity of the hydrodistilled (HD) and nanoemulsions of the *S. satureioides* essential oil in comparison with TROLOX and ascorbic acid. The absorbance was measured at 517 nm using a spectrophotometer (Cecil 2010, Cecil Instr. Ltd., Cambridge, UK) [33]. All tests were repeated three times, and the results were averaged.

Cupric reducing antioxidant capacity (CUPRAC) assay:CUPRAC was determined according to Bouratoua et al. [34]. The method includes mixing of 40 μL of sample solution with 50 μL of a copper(II) chloride solution, 50 μL of neocuproine alcoholic solution, and 60 μL of ammonium acetate aqueous buffer at pH 7. After 30 min, the absorbance was read at 450 nm.Results were recorded as absorbance compared with the absorbance of TROLOX and ascorbic acid, which were used as antioxidant standards.

The ABTS free radical–scavenging assay: The antioxidant activity of the HD oil and its nanoemulsion was determined by their ability to decolorize the radical cation (ABTS^•+^) as previously detailed [34]. The method comprises the addition of 180 μL of ABTS solution to 20 μL of sample solution in methanol at different concentrations. After 10 min, the percentage inhibition at 734 nm was calculated for each concentration relative to a blank absorbance (methanol). The scavenging capability of ABTS was calculated using the following equation:

ABTS^•+^ scavenging activity (%) = ((A_Control_ − A_Sample_)/A_Control_) × 100. Total phenolic content: The determination of the total phenolic contents in samples was conducted using the Folin–Ciocalteu reagent and gallic acid as standard, according to Singleton et al. [35] modified method. The absorbance measurement was performed at 765 nm on reaction mixtures incubated in a thermostat at 45 °C for 45 min.

Estimation of flavonoids content: The flavonoid content in essential oils and nanoemulsions was determined by the measurement of absorbance at 430 nm. Absorbance values depend on complex flavonoid–aluminum formation. Rutin was used to make the calibration curve, and then 1 mL of diluted samples was separately mixed with 1 mL of a 2% aluminum chloride methanolic solution. After incubation at room temperature for 15 min, the absorbance of the reaction mixture was measured at 430 nm, and the flavonoid content was expressed in mg per g of rutin equivalent (RE) [36].

### 3.7. Evaluation of the Cytotoxicity of the S. satureioides Coss. et Durieu Essential Oil and Its Nanoemulsions Against HepG2 and THLE2 Cells by the MTT Assay

Hep G2 and THLE2 were cultured at a density of 1 × 10^4^ cells/well (100µL) in a culture medium (DMEM) supplemented with antibiotics (10,000 U of penicillin and 10 mg of streptomycin in 0.9% saline) and 10% serum (FBS) and then incubated for 24 h at 37 °C and 5% CO_2_. After attachment for 24 h, a serially diluted essential oil and its nanoemulsions were applied at concentrations ranging from 1000 to 6.25 μg/mL for THLE2 and from 200 to 6.25μg/mL for HepG2 cells. A positive control (5-FU) at concentrations ranging from 400 to 5 μg/mL was applied in parallel. Subsequently, 10 μL of a 12-mM MTT stock solution (5 mg/mL MTT in sterile PBS) was added to each well. After incubation for 4 h at 37 °C, the MTT solution was eliminated, and the precipitated purple formazan crystal was dissolved in DMSO for 20 min. A negative control of 10 μL of the MTT stock solution was added to 100 μL of the uncultured medium. With an ELISA reader, the absorbance was measured at 570 nm. The curve was illustrated on the basis of the variation of the proportions of surviving cells [(OD of sample − OD of blank)/(OD of control − OD of blank) × 100%] according to concentrations, and IC_50_ was calculated using the sigmoidal curve obtained [26].

### 3.8. Statistical Analysis

Statistical Package for Social Sciences (SPSS) software version 16 was used for statistical analysis, and the data were analyzed using Student’s t-test and the analysis of variance (ANOVA) and expressed as mean ± SD.

## 4. Conclusions

The use of high-pressure homogenization to load the *Saccocalyx satureioides* Coss. et Durieu hydrodistilled oil in nanosystems has affected the volatile composition of the oil and its nanoemulsions and led to a difference in tested biological activities. It has a positive effect on antioxidant and anticancer activities, which makes this technique applicable and useful in several areas, such as pharmaceuticals, cancer treatment, and food biopreservation. The oxygenated terpenes were affected dramatically by such homogenization techniques, while an inverse relationship has been observed among different compounds.

## Figures and Tables

**Table 1:** Volatile constituents identified from the hydrodistilled (HD) and nanoemulsions of *S. satureioides* essential oil using GC-MS.

S/N	Compound	KI ^a^	% Area ^b^ *S. satureioides* Oil		Identification Method ^c,d^
			HD	Nanoemulsions	
1	Tricyclene	921	0.46 ± 0.03	n.d.	MS & KI
2	α-Thujene	928	0.56 ± 0.05	n.d.	MS & KI
3	α-Pinene	932	5.68 ± 0.06	n.d.	MS, KI& ST
4	Camphene	971	10.26 ± 0.11	n.d.	MS, KI& ST
5	β-Pinene	978	0.65 ± 0.12	0.97 ± 0.09	MS & KI
6	β-Myrcene	991	0.12 ± 0.02	0.89 ± 0.06	MS & KI
7	α-Terpinene	1004	0.72 ± 0.08	1.37 ± 0.13	MS, KI& ST
8	p-Cymene	1008	5.61 ± 0.33	6.99 ± 0.21	MS, KI& ST
9	Limonene	1029	2.74 ± 0.13	0.69 ± 0.04	MS, KI& ST
10	γ-Terpinene	1088	3.89 ± 0.08	10.7 ± 0.15	MS, KI& ST
11	Linalool	1089	0.26 ± 0.05	1.78 ± 0.30	MS, KI& ST
12	*trans*-Pinocarveol	1139	0.07 ± 0.01	n.d.	MS & KI
13	Camphor	1141	0.38 ± 0.02	n.d.	MS & KI
14	*cis*-Chrysanthenol	1145	0.08 ± 0.01	n.d.	MS & KI
15	Borneol	1148	25.71 ± 0.37	0.41 ± 0.02	MS, KI& ST
16	Terpinen-4-ol	1155	1.58 ± 0.09	1.42 ± 0.11	MS, KI& ST
17	α-Terpineol	1165	25.61 ± 0.27	1.60 ± 0.20	MS, KI& ST
18	Isobornyl formate	1225	0.53 ± 0.04	n.d.	MS & KI
19	Bornyl acetate	1263	0.53 ± 0.05	n.d.	MS & KI
20	Thymol	1267	12.8 ± 0.15	29.99 ± 0.33	MS, KI& ST
21	Carvacrol	1276	0.62 ± 0.05	41.27 ± 0.18	MS, KI& ST
22	α-Gurjunene	1392	0.11 ± 0.01	n.d.	MS & KI
23	β-Caryophyllene	1414	0.21 ± 0.02	0.58 ± 0.11	MS & KI
24	Aromadendrene	1435	0.08 ± 0.01	n.d.	MS & KI
25	Alloaromadendrene	1444	0.12 ± 0.03	n.d.	MS & KI
26	γ-Cadinene	1498	0.11 ± 0.07	n.d.	MS & KI
27	δ-Cadinene	1501	0.21 ± 0.08	n.d.	MS & KI
28	Spathulenol	1561	0.1 ± 0.06	n.d.	MS & KI
	Total	-	99.80	98.65	-

^a^ Confirmed by comparison with Kovats index on a DB5 column [16]. ^b^ Values represent averages ±standard deviations for triplicate experiments.^c^Confirmed by comparison with the mass spectrum of the authentic compound. ^d^ Identification by comparison with data obtained from the National Institute of Standards and Technology (NIST) mass spectra library.n.d: not detected.

**Table 2:** Activity of HD and nanoemulsions of *S. satureioides* essential oil in comparison to the antioxidant standards.

Material	IC_50_ (µg/mL) DPPH *	A_0.5_ (µg/mL) CUPRAC Assay *	IC_50_ (µg/mL) ABTS Assay *	Total Flavonoid Content * mg CE/g	Total Phenolic Content * mg RE/g
				for 1 mg/mL	for 1 mg/mL
*S. satureioides* HD oil	22.14^a^ ± 0.12	37.72^a^ ± 0.31	11.39^a^ ± 0.05	0.401^a^ ± 0.02	0.863^a^ ± 0.02
Nanoemulsions of *S. satureioides* oil	47.87^b^ ± 0.21	754.67^b^ ± 3.06	257.86^b^ ± 3.71	0.302^b^ ± 0.01	0.656^b^ ± 0.03
TROLOX **	5.12^c^ ± 0.21	8.69^c^ ± 0.14	3.21^c^ ± 0.06	-	-
Ascorbic acid **	4.39^d^ ± 0.01	8.31^d^ ± 0.15	3.04^d^ ± 0.05	-	-

* Values represent averages ±standard deviations for triplicate experiments. Means with the same superscript letter within the same column are not significantly different (*p* > 0.05).** Antioxidant standards.
